# Luteinizing hormone stimulates the expression of amphiregulin in human theca cells

**DOI:** 10.1186/s13048-022-01062-5

**Published:** 2022-12-07

**Authors:** Yang Liu, Yiping Zhong, Xiaoting Shen, Xi Guo, Rihan Wu, Turui Yang, Minghui Chen

**Affiliations:** 1 Reproductive Medicine Center, The First Affiliated Hospital of Sun Yat-sen University, Zhoushan 2 Road, Guangzhou, Guangdong People’s Republic of China; 2 Guangdong Provincial Key Laboratory of Reproductive Medicine, The First Affiliated Hospital of Sun Yat-sen University, Guangzhou, China; 3 Obstetrics and Gynaecology Hospital of Fudan University, Shanghai, China

**Keywords:** Amphiregulin, Theca cells, Granulosa cells, Luteinizing hormone, cAMP/PKA signalling pathway

## Abstract

**Background:**

Luteinizing hormone (LH) can stimulate mural granulosa cells to produce Amphiregulin (AREG), which can induce the resumption of meiosis in oocytes. Theca cells are present in the outer layer of follicles, providing communication with the pituitary axis through the established vascular system around the follicle. As LH target cells, it is unknown whether theca cells can produce AREG after LH stimulation.

**Methods:**

Primary cultured human theca cells were treated with LH (with or without the inhibitor of PKA, H89), or agonists of adenylate cyclase (forskolin or db-cAMP). The mRNA and protein levels of *AREG* were evaluated by RT-qPCR, immunochemistry, immunofluorescence, western blotting, and ELISA.

**Results:**

Immunohistochemistry of normal ovarian tissue obtained in the early-mid follicle phase showed that *AREG* expression was absent in both the theca layer and the granulosa cell layer of antral follicles. Double immunofluorescent staining revealed colocalization of AREG and CYP17A1 in human theca cells and colocalization of FSHR and AREG in human granulosa cells isolated from follicular fluid collected during IVF/ICSI after hCG trigger. LH significantly increased the mRNA and protein levels of *AREG* in human theca cells and the concentration of AREG in the culture medium. Forskolin and db-cAMP, activators of the cAMP/PKA signalling pathway, also significantly increased the mRNA and protein levels of *AREG* in human theca cells and the concentration of AREG in the culture medium. H89 antagonized the stimulating effect of LH on *AREG* expression in human theca cells. In addition, the concentration of AREG was lower in polycystic ovarian syndrome (PCOS) follicular fluid than in normal follicular fluid. The mRNA levels of *AREG* were significantly lower in PCOS granulosa cells and theca cells than in normal granulosa cells and theca cells.

**Conclusion:**

LH can stimulate the expression of *AREG* in human theca cells, and the adenylate cyclase/cAMP/PKA cascade may mediate this process. Expression of *AREG* is decreased in PCOS theca cells compared to normal theca cells, with or without LH stimulation.

## Background

The mid-cycle luteinizing hormone (LH) surge activates multiple signalling networks in the ovarian follicle [1], which then result in the resumption of meiosis, oocyte maturation, cumulus dilatation, rupture of the follicle, and release of the oocyte during ovulation [2–4]. However, cumulus cells and oocytes express very few LH receptors and do not respond to direct exposure to LH. LH exerts its effect on cumulus-oocyte complexes (COCs) via paracrine factors secreted by mural granulosa and theca cells, which express LH receptors [5].

Amphiregulin (AREG), a member of the epidermal growth factor (EGF) family, is involved in the regulation of cell survival, proliferation, and movement by activating intracellular signals via its EGF receptor (EGFR) [6]. AREG serves as a critical intermediate between mural cells and COCs following the mid-cycle LH surge. AREG can induce oocyte meiosis resumption by inhibiting the expression of *CNP* in granulosa cells and *NPR2* in cumulus cells and reducing the production of cGMP, which can be antagonized by EGFR inhibitors [7, 8]. LH can stimulate mural granulosa cells to secrete AREG, which activates EGFR signalling in cumulus cells [9], leading to a decrease in cGMP synthesis in response to NPR2 inactivation [8]. Meanwhile, LH increases the cGMP-hydrolytic activity of PDE5 dependent on PKA signalling [10]. Finally, the decrease of cGMP results in cumulus expansion and oocyte maturation [4].

Theca cells present in the outer layer of follicles provide communication with the pituitary axis through the established vascular system around the follicle. LH can activate LH receptors and stimulate androgen production in theca cells [11]. However, it is unclear how theca cells transmit ovulatory signals into the follicle. A study of equine preovulatory follicles reported that hCG can stimulate *AREG* expression in theca cells [12]. Because LH and hCG share the same receptor, namely, the luteinizing hormone/choriogonadotropin receptor (LHCGR), these results indicated that theca cells may transmit LH signals to inner follicle cells by secreting AREG.

Polycystic ovarian syndrome (PCOS) is the most common endocrinopathy affecting women of reproductive age. Elevated LH concentrations, which reflect an increase in LH pulse frequency and amplitude, have been observed in up to 75% of women with PCOS [13]. Elevated LH concentrations could stimulate the secretion of AREG in granulosa cells. However, *AREG* was reported to be downregulated in PCOS follicular fluid granulosa cells and cumulus cells [14–16]. Whether *AREG* is also downregulated in PCOS theca cells is still unknown.

In the present study, we aimed to investigate the expression of *AREG* in human theca cells and the regulation of *AREG* expression in human theca cells by LH. Subsequently, we observed the expression pattern of *AREG* in PCOS theca cells.

## Materials and methods

### Ethical and participants

This study was approved by the Medical Ethics Committee of the First Affiliated Hospital of Sun Yat-sen University, and written informed consent was obtained from each patient included. PCOS was diagnosed according to the Rotterdam criteria 2003 [17]. In the experiments involving PCOS, the theca cells and granulosa cells in both the PCOS group and the control group were collected from patients with gonadotropin-releasing hormone (GnRH) antagonist protocol using GnRH agonist (0.2 mg) and human chorionic gonadotropin (hCG, 2000 IU) to induce oocyte maturation. In the other experiments, the theca cells and granulosa cells were collected from patients with GnRH agonist long protocol or GnRH antagonist protocol using hCG (10000 IU) to induce oocyte maturation.

### Cell culture

For each experiment, follicular fluid was collected during oocyte retrieval from 10 to 20 patients undergoing IVF/ICSI and pooled. The same batch of follicular fluid was used for different groups in the same experiment. Different batches of follicular fluid were used in biological replicates. The inclusion criteria were as follows: under 40 years of age, basal serum follicle-stimulating hormone (FSH) < 10 IU/L, and body mass index (BMI) < 35 kg/m^2^. The diagnosis of PCOS status was based on the Rotterdam criteria [17]. The exclusion criteria were endometriosis, other endocrine diseases, a history of radiotherapy or chemotherapy, poor ovarian response, or ≥ 3 IVF/ICSI cycles.

For the isolation of theca cells, the follicular fluid was centrifuged at 751 × g for 10 min at room temperature. The precipitate was reconstituted in phosphate-buffered saline (PBS) and then filtered by a 100 μm cell strainer (BD Biosciences, Franklin Lakes, NJ, USA). The ovarian tissue was repeatedly blown with PBS during the filtration process to remove the granulosa cells. The next steps were performed according to the previously described procedure [18]. Briefly, the ovarian tissues that remained on the strainer were collected and digested with 5 mg/mL type I collagenase (Sigma, St. Louis, MO, USA) solution at 37 ℃ for approximately 90 min and pipetted every 15 min to speed up the digestion process. The dispersed cells were collected every 15 min, washed with PBS twice, and resuspended in PBS. Undigested tissue was then removed by a 40 μm cell strainer (BD Biosciences, Franklin Lakes, NJ, USA).

For the isolation of mural granulosa cells, follicular fluid was collected after filtration for ovarian tissue with a 100 μm cell strainer and was centrifuged at 751 × g for 8 min. Granulosa cells were purified using 50% Percoll (Sigma, St. Louis, MO, USA) through gradient centrifugation for 15 min at 563 × g. Ovarian tissue fragments were then removed again from the granulosa cell suspension with a 40 μm cell strainer (BD Biosciences, Franklin Lakes, NJ, USA).

The theca cells and granulosa cells were from the same pools in the experiments that required both theca cells and granulosa cells. The theca cells and granulosa cells from PCOS patients were isolated and purified as the method mentioned above.

The dispersed theca cells and granulosa cells were washed with PBS and resuspended in Dulbecco’s modified Eagle’s medium (DMEM)/F12 medium supplemented with 10% foetal bovine serum (FBS) (Gibco, CA, USA), 100 U/mL penicillin, and 100 µg/mL streptomycin (Gibco, CA, USA). The purified cells were counted with a haemocytometer, and the cell viability was determined by trypan blue staining. Cells were plated on six-well culture dishes at a density of 3 × 10^5^ cells/well and cultured at 37 °C in a humidified incubator containing 5% CO_2_, and the medium was changed daily. After 48 h of culture, cells were treated with 400 mIU/ml LH and cultured for 0 (control), 3, 6, 12, or 24 h or with various doses of LH (0, 50, 100, 200, 400, or 800 mIU/ml), 10 µM forskolin, 500 µM db-cAMP, or 400 mIU/ml LH with 10 µM H89 (at the same time as the stimulus) for 6 h. Recombinant LH (Luveris®, Merck Serono S.A.) was dissolved with saline. The LH treatment group and the control group were added the same amount of saline.

### Immunohistochemistry (IHC)

Paraffin sections of normal ovarian tissue were obtained from patients who had undergone bilateral salpingo-oophorectomy in the early-mid follicle phase with or without hysterectomy for a uterine malignant tumour before chemotherapy or radiotherapy. Ovarian tissues from follicular fluid collected during oocyte retrieval in IVF/ICSI were fixed in 4% paraformaldehyde (PFA) overnight. Then, the samples were dehydrated and embedded in paraffin, and sectioned at 4-µm thickness. After deparaffinization, antigen retrieval, and blocking in 5% BSA, the slides were incubated overnight at 4 ℃ in rabbit anti-human antibodies against LHCGR (1:200, ab125214, Abcam, Cambridge, UK), cytochrome P450 family 17 subfamily A member 1 (CYP17A1) (1:250, ab134910, Abcam, Cambridge, UK), or AREG (1:200, ab234750, Abcam, Cambridge, UK). Slides were washed and incubated with horseradish peroxidase (HRP)-labelled goat anti-rabbit IgG secondary antibodies (1:200, GB23303, Servicebio, Wuhan, Hubei, China) for 50 min. The antibody complex was detected by a DAB reagent (G1211, Servicebio, Wuhan, Hubei, China). Control experiments included samples treated in the same manner, but normal rabbit IgG was used instead of the primary antibodies. The sections were counterstained with haematoxylin.

### Immunofluorescence analysis

Cells were placed on cover glasses (801007, NEST, Wuxi, Jiangsu, China) at a density of 6 × 10^4^ cells/well in 24-well plates, culturing at the former mentioned condition for 48 h. Glasses/cells were fixed in 4% paraformaldehyde (PFA) for 30 min at 4 ℃ and washed three times with PBS containing 0.1% Tween-20 (PBST). Subsequently, 0.5% Triton X-100 (Solarbio, Beijing, China) was used to permeabilize the theca cells for 15 min except for those used for staining for follicle-stimulating hormone receptors (FSHR). Next, PBST containing 5% donkey serum (Santa Cruz Biotechnology, CA, USA) was added to block nonspecific binding sites for 30 min. Rabbit anti-human CYP17A1 primary antibody (1:200, 94,004 S, CST, Danvers, MA, USA), rabbit anti-human FSHR primary antibody (1:200, ab113421, Abcam, Cambridge, UK), goat anti-human AREG primary antibody (1:200, AF262-SP, R&D Systems, Inc., Minneapolis, MN, USA), or PBS (as a negative control) was then added to the respective wells and incubated overnight at 4 ℃. After rinsing in PBST three times, glasses/cells were incubated with Alexa Fluor 488-conjugated donkey anti-rabbit secondary antibodies (1:200, ab150073, Abcam, Cambridge, UK) and Alexa Fluor 647-conjugated donkey anti-goat secondary antibodies (1:200, ab150131, Abcam, Cambridge, UK) for 1 h at 37 °C. After three washes with PBS, the nuclei were stained with DAPI (Aquarius/Cytocell Ltd., Cambridge, UK). The cells were visualized using a fluorescence microscope (DMI8, Leica, Wetzlar, German).

### RNA extraction, reverse transcription, and real-time quantitative PCR (RT–qPCR)

We extracted RNA with the GeneJET RNA Purification Kit (Thermo Scientific™, Waltham, MA, USA) according to the manufacturer’s instructions and quantified the RNA by A260/280 ultraviolet spectrophotometry (NanoPhotometer, Implen Inc., CA, USA). Approximately 500 ng to 1 µg total RNA was subjected to reverse transcription (RT) with PrimeScript™ RT Master Mix (RR036A, Takara Bio Inc., Japan) following the instructions. The differential expression of *AREG* (Hs00950669_m1), *LHCGR* (Hs00174885_m1), and *EGFR* (Hs01076090_m1) mRNA in theca cells was quantified using TaqMan Gene Expression Assays (Applied Biosystems, Life Technologies, Franklin Lakes, NJ, USA). Quantification was achieved by the Roche LightCycler 480 machine using Premix Ex Taq (Probe qPCR) (RR390A, Takara Bio Inc., Otsu, Shiga, Japan). The relative mRNA level was calculated by the 2^−ΔΔCT^ method using *GAPDH* (Hs02786624_g1) as a reference gene.

#### AREG ELISA

The cell lysate samples, culture medium, and follicular fluids were frozen at -20 °C for subsequent measurement of AREG by the Human Amphiregulin Quantikine ELISA Kit (DAR00, R&D Systems, Inc. Minneapolis, MN, USA) according to the manufacturer’s instructions. The detection limit of AREG was 3.56 pg/mL. Cells were cultured at a density of 3 × 10^5^ cells/well for AREG secretion experiments. LH can stimulate the proliferation of theca cells. To eliminate the interference of cell proliferation after LH treatment on AREG secretion analysis, the AREG concentrations were shown as the actual concentration measured divided by the total number of cells which unit was pg/10^4^ cells.

### Western blot

Cells were lysed with Cell Lysis Buffer (CST, Danvers, MA, USA). A total of 20 µg of total protein was separated by 10% SDS PAGE and transferred to 0.42 μm PVDF membranes (Millipore, Corp, Billerica, MA, USA). Five per cent non-fat dry milk in Tris-buffered saline (TBS) containing 0.1% Tween-20 (TBST) was used to block nonspecific binding. Then, the blots were incubated with primary antibodies against AREG (1:1000, Proteintech, 16036-1-AP) or GAPDH (1:3000, 5174 S, CST, Danvers, MA, USA) overnight at 4 °C. After being washed four times with TBST, blots were incubated with an HRP-conjugated secondary antibody (1:3000, 7074 S, CST, Danvers, MA, USA) for 1 h. After being extensively washed with TBST, blots were reacted with chemiluminescent HRP substrate (Millipore, Corp, Billerica, MA, USA) and detected by ChemiDoc Touch (Bio–Rad Laboratories, Inc., Hercules, CA, USA). Images were analysed by ImageJ software (National Institutes of Health, Bethesda, MD, USA).

### Statistical analysis

Statistical analysis was conducted using SPSS 22 (IBM Corporation, Armonk, NY, USA). The Kolmogorov–Smirnov test was applied to analyse the distribution of data using a two-group comparison. The results are described as the mean ± standard error of the mean (SEM) of replicate samples. Between-group comparisons were performed by Student’s *t* test if the data were normally distributed or by the Wilcoxon test otherwise. Differences among multiple groups were assessed by ANOVA followed by Tukey’s multiple-comparison post hoc test if the equal variance was assumed or by Dunnett’s T3 post hoc test otherwise. *P* < 0.05 was considered statistically significant.

## Results

### Identification of human theca cells

**Fig. 1:**
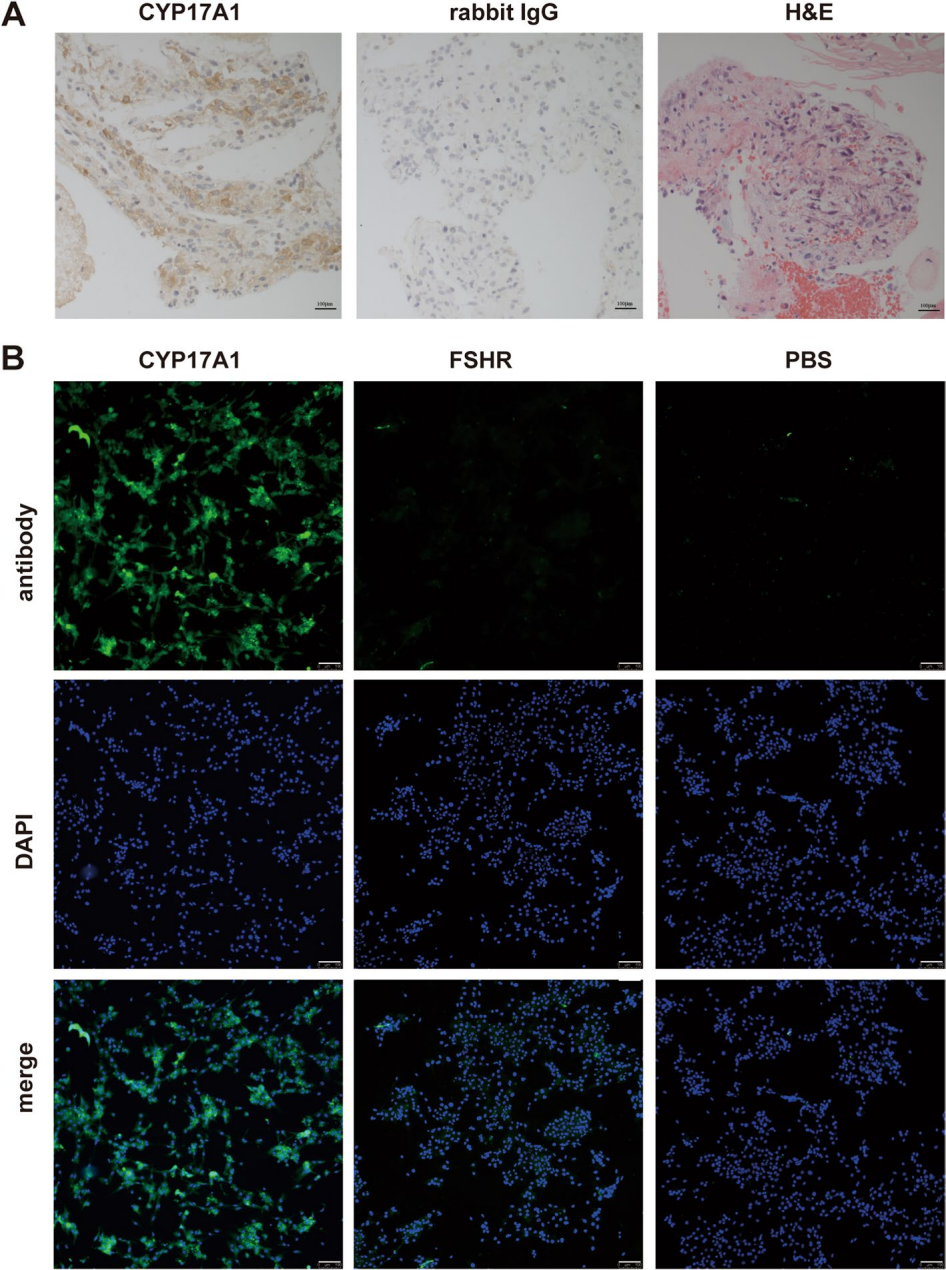
Identification of human theca cells. **A** Representative images of IHC staining for CYP17A1 in ovarian tissues collected from follicle fluid during oocyte retrieval. In the control group, samples were treated in the same manner, but normal rabbit IgG was used instead of the primary antibodies. Scale bar: 100 μm. **B** Representative images of IF staining for CYP17A1 (green) and FSHR (green) in the isolated cells. In the control group, samples were treated in the same manner, but PBS was used instead of the primary antibodies. Nuclei were stained with DAPI (blue). Scale bar: 250 μm

Immunohistochemical staining of the theca cell marker CYP17A1 was observed in ovarian tissues collected from follicular fluid during oocyte retrieval. The cells isolated from these CYP17A1-positive ovarian tissues were then subjected to immunofluorescence staining; 93 ± 3% of the cells were positive for CYP17A1, and 0% of the cells were positive for FSHR (Fig. 1).

### Expression of AREG is absent in antral follicles in the early-mid follicle phase

**Fig. 2:**
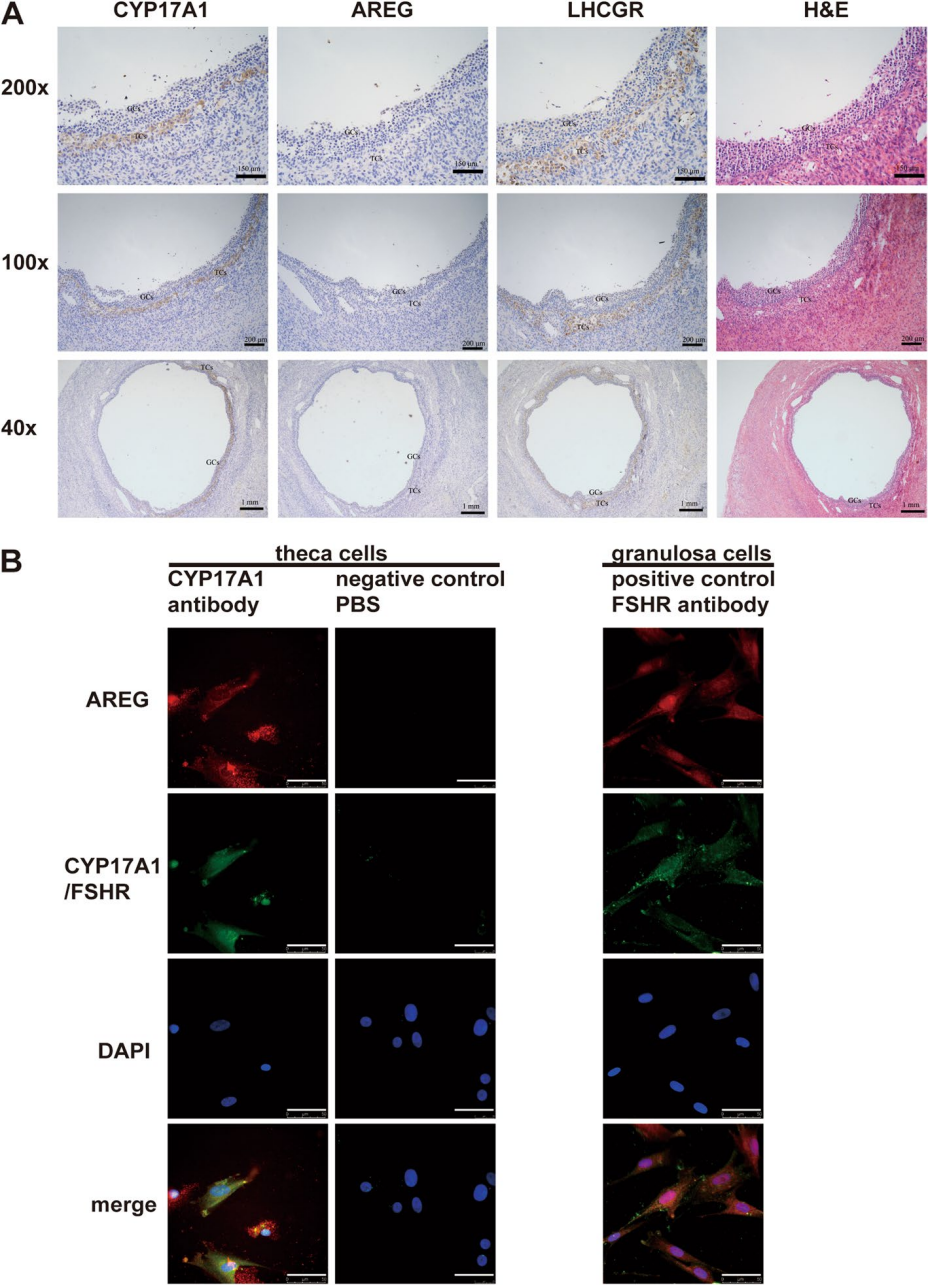
AREG in the human ovary. **A** Representative images of IHC staining of AREG, CYP17A1, and LHCGR in the early-mid follicle phase. Scale bar: 150 μm, 200 μm, 1 mm; GCs, granulosa cells; TCs, theca cells. **B** Representative images of double immunofluorescent staining of AREG (red) and CYP17A1 (green), or AREG (red) and FSHR (green) in human theca cells and granulosa cells isolated in follicular fluid during oocyte retrieval after hCG trigger. Nuclei were stained with DAPI (blue). Scale bar: 50 μm

Immunohistochemistry of normal ovarian tissue obtained in the early-mid follicle phase showed that AREG protein was absent, while CYP17A1 and LHCGR were strongly detected in the theca cell layer of antral follicles. AREG was also not detected in the granulosa cell layer of antral follicles (Fig. 2A).

### AREG is expressed in human theca cells and granulosa cells after the hCG trigger

Human theca cells and granulosa cells were isolated from follicular fluid obtained during oocyte retrieval after the hCG trigger. Double immunofluorescent staining revealed colocalization of AREG and CYP17A1 in human theca cells and colocalization of FSHR and AREG in human granulosa cells (Fig. 2B).

### LH stimulates AREG expression in human theca cells

**Fig. 3:**
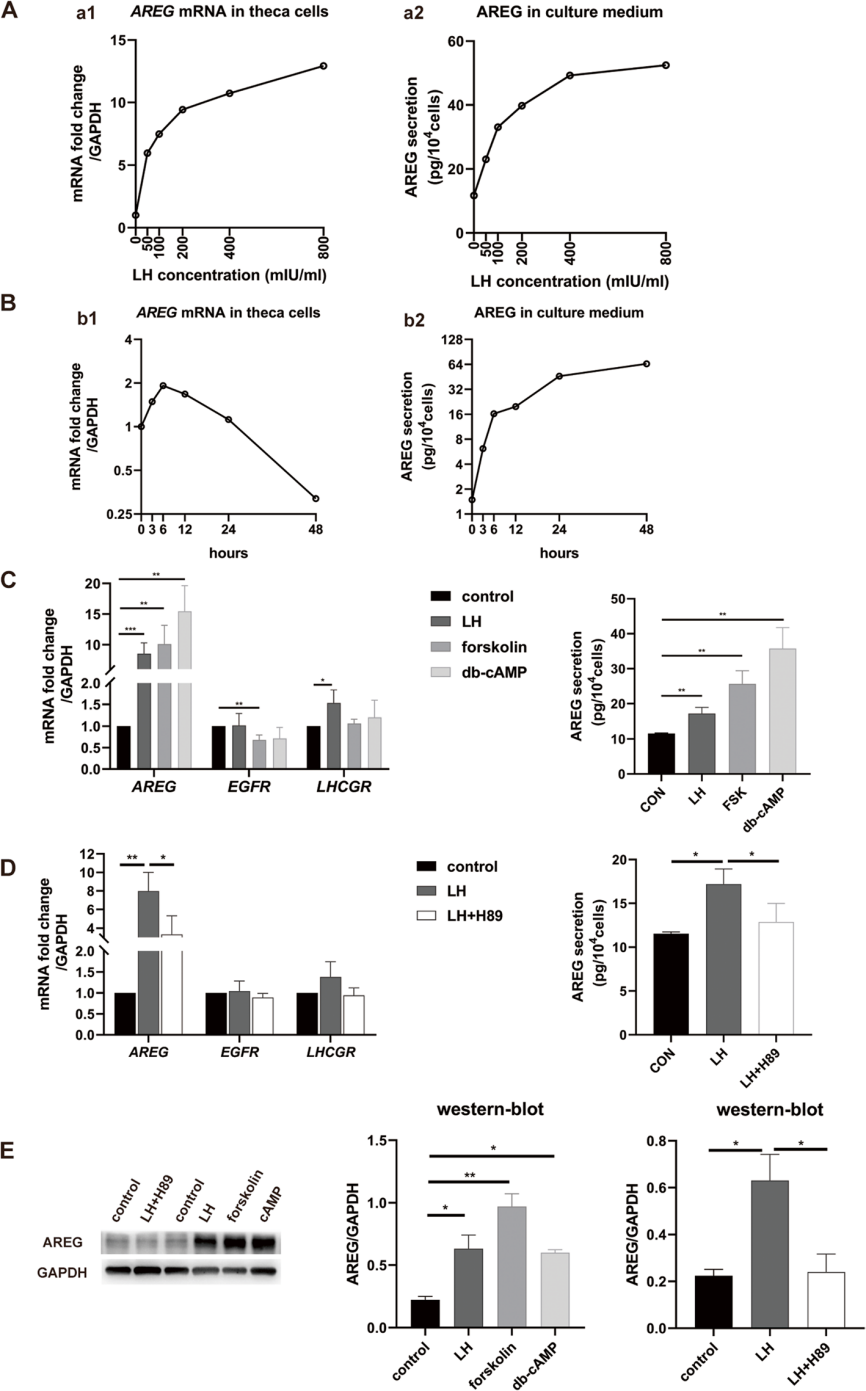
The effect of LH on *AREG* expression in human theca cells and the role of the cAMP/PKA signalling pathway in the process. **A** LH stimulated both the gene expression of *AREG* in theca cells (a1) and the secretion of AREG in theca cell culture medium (a2) in a dose-dependent manner. Cells were cultured for 6 h with or without different concentrations of LH. **B** Time course of the expression of AREG in the presence of LH (400 mIU/mL) in human theca cells. The mRNA levels of *AREG* were measured by quantitative PCR (b1); the levels of AREG protein in the culture medium were measured by ELISA (b2). **C**, **D**, and **E** Relative expression of *AREG*, *EGFR*, and *LHCGR* in human theca cells treated with LH (400 mIU/mL, with or without 10µM H89), forskolin (10 µM), or db-cAMP (500 µM) for 6 h. The mRNA levels of *AREG* were measured by quantitative PCR; the levels of AREG protein in the culture medium were measured by ELISA (C and D); the levels of AREG protein in theca cells were measured by western blot (D). In A and B, each experiment was performed once. In C-E, each experiment was repeated three times. *P < 0.05, ** *P* < 0.01, ***P < 0.001

Treatment with LH stimulated both the gene expression of *AREG* in theca cells and the secretion of AREG in theca cell culture medium in a dose-dependent manner. The increase in the mRNA and protein levels of *AREG* reached a plateau when the LH concentration was 400 mIU/ml (Fig. 3A). After treatment with 400 mIU/ml LH, the mRNA level of *AREG* increased rapidly and peaked at 6 h posttreatment and then subsequently declined to the baseline level at 24 h posttreatment (Fig. 3B). The level of AREG protein in the culture medium gradually increased after treatment with 400 mIU/ml LH and reached a plateau at 24 h posttreatment.

**The cAMP/PKA signalling pathway is involved in the regulation of AREG expression by LH in human theca cells**.

LH significantly increased the mRNA and protein levels of *AREG* in human theca cells and the concentration of AREG in the culture medium. Forskolin and db-cAMP, activators of the cAMP/PKA signalling pathway, also significantly increased the mRNA and protein levels of *AREG* in human theca cells and the concentration of AREG in the culture medium (Fig. 3C, E). LH stimulated *LHCGR* expression but did not affect *EGFR* expression. Forskolin suppressed the expression of *EGFR* but did not affect the expression of *LHCGR*. Treatment with db-cAMP did not affect the expression of *EGFR* or *LHCGR* (Fig. 3C-D). H89, an inhibitor of PKA, antagonized the stimulating effect of LH on the mRNA and protein levels of *AREG* in theca cells and the concentration of AREG in the culture medium but had no significant effect on the mRNA level of *EGFR* or *LHCGR* (Fig. 3D, E).

### Expression of AREG in PCOS theca cells and granulosa cells

**Fig. 4:**
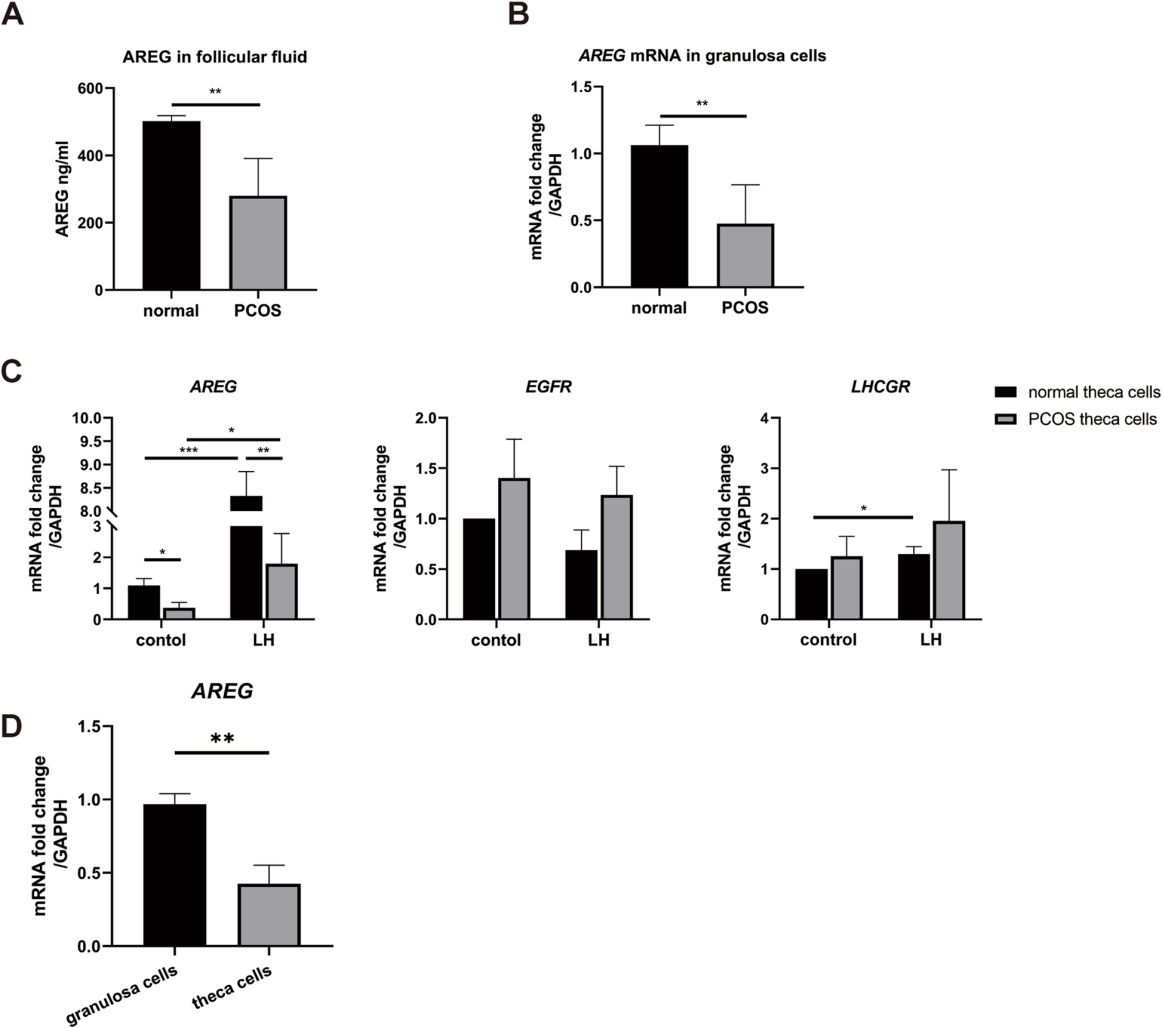
Expression of *AREG* in PCOS granulosa cells and theca cells. **A** The concentration of AREG in follicle fluid of PCOS patients and normal participants were measured by ELISA. **B** The mRNA levels of *AREG* in granulosa cells of PCOS patients and normal participants were measured by quantitative real-time PCR. **C** The mRNA levels of *AREG, EGFR*, and *LHCGR* in theca cells of PCOS patients and normal participants cultured for 6 h with or without the presence of LH (400 mIU/mL) were measured by quantitative real-time PCR. **D** The mRNA levels of *AREG* in both granulosa cells and theca cells were measured by quantitative real-time PCR. In A and B, data are displayed as mean ± SEM (n = 6 per group); in C and D, each experiment was repeated three times. *P < 0.05, ** *P* < 0.01, ***P < 0.001

The concentration of AREG was lower in PCOS follicular fluid than in normal follicular fluid (Fig. 4A). The mRNA levels of *AREG* were significantly lower in PCOS granulosa cells than in normal granulosa cells (Fig. 4B). The mRNA levels of *AREG* were also significantly lower in PCOS theca cells than in normal theca cells (Fig. 4C). After treatment with LH, the mRNA levels of *AREG* increased in both PCOS and normal theca cells, while the mRNA level of *AREG* was lower in the PCOS group than in the normal group in the presence of LH (Fig. 4C). Besides, the level of *AREG* mRNA in theca cells was significantly lower than that in granulosa cells (Fig. 4D). Treatment with LH did not affect the mRNA level of *EGFR* or *LHCGR* in PCOS or normal theca cells, and there were no significant differences in the mRNA level of *EGFR* or *LHCGR* between the PCOS group and the normal group.

## Discussion

In this study, we extracted human theca cells from tissue fragments in follicular fluid obtained during oocyte retrieval in IVF. Immunohistochemical staining of the theca cell marker CYP17A1 [19] observed in the tissue fragments in follicular fluid indicated the tissue fragments contained a theca layer. Immunofluorescence staining of FSHR which is the marker of granulosa cells [20] is absent in these isolated cells indicating a low probability of granulosa contamination.

AREG is a critical factor that transmits the ovulatory signal of the LH surge to the COCs. The results of previous studies suggested that the LH surge stimulated granulosa cells to produce AREG and then induced oocyte meiosis resumption by regulating the production of cGMP in cumulus cells [7, 8]. The theca cell layer is the outer layer of the ovarian follicle, and LH receptors are mainly distributed in the theca cell layer, as our results showed. Therefore, theca cells may be the first signal transfer site of LH signalling in ovarian follicles. A previous study of equine cells found that after treatment with hCG, which shares the same receptor with LH, *AREG* mRNA significantly increased in both theca cells and granulosa cells [12]. Our results showed that AREG is undetectable in theca cells of human antral follicles before the LH surge. Nevertheless, AREG is expressed in theca cells of mature ovarian follicles after the hCG trigger. Moreover, LH stimulated AREG production in human theca cells that had been exposed to hCG. The above evidence suggests theca cells from mature ovarian follicles may play a role in transmitting the LH signal into ovarian follicles; that is, after LH stimulation, the theca cells produce AREG, which can induce oocyte meiosis resumption. Nevertheless, it is still unknown whether theca cells from the early-mid stage of the ovarian follicles respond to LH stimulation since discarded human ovarian tissue is difficult to obtain after gynaecological surgery.

LH receptor coupling with the adenylate cyclase/cAMP/PKA cascade is well documented in mammals [21, 22]. Khampoun Sayasith found that adenylate cyclase, activated by forskolin, increased *AREG* mRNA levels in bovine granulosa cells in vitro, indicating that adenylate cyclase/cAMP may be involved in the regulation of *AREG* expression [12]. In this study, we found that both forskolin and db-cAMP can significantly increase the expression of *AREG*, and the PKA inhibitor H89 significantly antagonizes the stimulating effect of LH on the expression of *AREG*. The degree of antagonism of H89 to LH-stimulated AREG expression was different at the protein level and mRNA level in this study. It may result from different sensitivity of detection techniques. The qPCR technique for the detection of mRNA level has high sensitivity while Western blot for the detection of protein level is a semi-quantitative analysis. Our results indicate that the adenylate cyclase/cAMP/PKA cascade may mediate the regulation of *AREG* expression by LH.

An increase in LH secretion with normal FSH secretion has been widely accepted as a specific endocrine profile of PCOS [13]. Elevated LH concentrations in PCOS could stimulate the secretion of AREG in granulosa cells, while *AREG* is downregulated in PCOS follicular fluid, granulosa cells, and cumulus [14–16]. The present study showed that expression of *AREG* decreased in PCOS granulosa cells compared to normal granulosa cells. Moreover, the expression of *AREG* was decreased in PCOS theca cells compared to normal theca cells, with or without LH stimulation. Furthermore, we found that the expression of *LHCGR* was similar in the PCOS group and normal group, regardless of LH stimulation, indicating that the downregulation of *AREG* in PCOS ovarian cells may not be caused by the decreased expression of *LHCGR*, which mediates the effect of LH. The underlying mechanism of the downregulation of *AREG* expression in PCOS theca cells and granulosa cells needs to be further explored. 

## Conclusion

In conclusion, LH can stimulate the expression of *AREG* in human theca cells, and the adenylate cyclase/cAMP/PKA cascade may mediate this process. Expression of *AREG* is decreased in PCOS theca cells compared to normal theca cells, with or without LH stimulation.

## Data Availability

All data underlying this article are available on reasonable request to the corresponding author.

## Abbreviations

LH: luteinizing hormone
AREG: Amphiregulin
CNP: natriuretic peptide precursor type C
PCOS: polycystic ovarian syndrome
COCs: cumulus-oocyte complexes
EGF: epidermal growth factor
EGFR: EGF receptor
LHCGR: luteinizing hormone/choriogonadotropin receptor
FSH: follicle-stimulating hormone
CYP17A1: cytochrome P450 family 17 subfamily A member 1
